# Antiseptic Cologne

**Published:** 1886-08-20

**Authors:** 


ANTISEPTIC COLOGNE.—Our attention has been invited to a preparation which is likely to prove of great service to the practitioner. It is Antiseptic Cologne, manufactured by Parke, Davis & Co., which combines the properties of an active disinfectant with those of a refreshing and agreeable perfume. The active constituents of this preparation are thymol, oil of eucalyptus and mercuric chloride, combined with a cologne of superior quality. The utility of this preparation is at once apparent. Nearly all the disinfectants in common use which have any real value, are limited in their uses about the house, and especially in the sick room, by their disagreeable odor. In the sick room this preparation may be employed in the form of a spray with an ordinary perfume atomizer, to overcome disagreeable odors. Of course, this must be merely as a paliative ; the air must be kept pure besides by free ventilation. It would seem to be adapted to meet several important indications when attending patients suffering from infectious diseases. It must be useful as a disinfectant for the hands in cases of infectious diseases or to remove the odor of Labarraque's Solution which is often employed for the same purpose. In gynecological and obstetric practice, such a preparation as this would seem to be of especial service. We learn that it is put up in half-pint bottles, labeled with full directions for use, which are sold for $1 each, or sample bottle, trial size, 25 cents.

